# Intención de abandono de la profesión enfermera por salud mental en Navarra (España) durante la pandemia de COVID-19

**DOI:** 10.23938/ASSN.1110

**Published:** 2025-04-30

**Authors:** Cristina García-Vivar, Marta Ferraz-Torres, Paula Escalada-Hernández, Nelia Soto-Ruiz

**Affiliations:** 1 Universidad Pública de Navarra (UPNA) Departamento de Ciencias de la Salud Pamplona España; 2 Instituto de Investigación Sanitaria de Navarra (IdiSNA) Pamplona España

**Keywords:** Pandemia de COVID-19, Enfermeras, Satisfacción laboral, Salud Mental, Encuestas y cuestionarios, Pandemic COVID-19, Nurses, Job satisfaction, Mental Health, Surveys and Questionnaires

## Abstract

**Fundamento:**

El objetivo es estimar la intención de abandono de la profesión enfermera o del puesto de trabajo en la comunidad foral de Navarra (España) durante la sexta ola de la pandemia por COVID-19 (octubre 2021-marzo 2022) y analizar su relación con el estado de salud mental de las enfermeras y sus características socio-demográficas.

**Metodología:**

Estudio descriptivo y transversal con enfermeras que trabajaron durante la pandemia de COVID-19 en instituciones sanitarias de Navarra. Se diseñó un cuestionario con escalas validadas para evaluar depresión, ansiedad, insomnio, estrés postraumático y la intención de abandonar el puesto de trabajo o la profesión enfermera. El cuestionario se envió por correo electrónico a las enfermeras colegiadas, cerrando la recogida de datos al alcanzar el tamaño muestral requerido.

**Resultados:**

Se recibieron 691 cuestionario completos. El 43% de las enfermeras manifestaron intención de abandonar la profesión y, de ellas, el 52% también consideraron la posibilidad de cambiar de puesto de trabajo. Las enfermeras con niveles moderados de ansiedad y estrés postraumático mostraron mayor intencionalidad de abandono de la profesión, así como aquellas con menor experiencia profesional.

**Conclusiones:**

La intención de abandono de la profesión enfermera en Navarra durante la sexta ola de la pandemia por COVID-19 se asocia significativamente con niveles moderados de ansiedad y estrés postraumático, y menor experiencia profesional. Es necesario implementar estrategias para mejorar las condiciones laborales, promover el bienestar mental y reducir el riesgo de abandono profesional de las enfermeras, especialmente en contextos de crisis sanitaria.

## Abstract (en)

**Background:**

This study aimed to assess the intention to leave the current job or the nursing profession among nurses in the Foral Community of Navarre (Spain) during the sixth wave of the COVID-19 pandemic (October 2021-March 2022). The research also evaluated associations between this intention and nurses’ mental health status and sociodemographic characteristics.

**Methods:**

Descriptive, cross-sectional study that included nurses who worked in healthcare institutions in Navarre during the COVID-19 pandemic. A questionnaire was designed using validated scales to assess depression, anxiety, insomnia, post-traumatic stress, and the intention to leave the job or profession. The questionnaire was distributed via e-mail to all registered nurses, and data collection concluded upon reaching the required sample size.

**Results:**

Of the 693 nurses who participated, 691 completed the questionnaire in full. Overall, 43% of respondents reported an intention to leave the nursing profession, and of these, 52% also considered leaving the current job. Nurses experiencing higher levels of anxiety and post-traumatic stress, as well as those with fewer years of professional experience, were significantly more likely to report an intention to leave.

**Conclusions:**

A substantial proportion of nurses in Navarre considered leaving the profession during the sixth wave of the COVID-19 pandemic, with mental health -particularly anxiety and post-traumatic stress- and limited professional experience being the key contributing factors. These findings underscore the need for targeted interventions to support nurses’ mental health, improve working conditions, and mitigate job abandonment during and after public health emergencies.

## INTRODUCCIÓN

Desde el comienzo de la pandemia por COVID-19, en 2020, se han confirmado más de 775 millones de nuevos casos y unos 7 millones de muertes en todo el mundo hasta junio de 2024^1^. En Europa 2,2 millones de personas han muerto a causa de la enfermedad^1^. España, uno de los países europeos en los que el COVID-19 tuvo un mayor impacto en sus inicios, registró un total de 13,9 millones de afectados y 121.760 fallecidos, según los últimos datos disponibles a junio de 2023^2^. Estos datos reflejan una clara afectación social de la pandemia de COVID-19 y una crisis sanitaria mundial sin precedentes, que ha tenido un gran impacto en los sistemas sanitarios^3^. El incremento de la prevalencia de la enfermedad y la creciente demanda sanitaria resultaron en una saturación de los hospitales^4^ y en una intensa presión asistencial en los servicios de atención primaria, hospitalaria y sociosanitaria^5^.

La sobrecarga en los servicios de salud ha dejado una profunda huella en los profesionales sanitarios implicados en su gestión y desarrollo, afectando de manera especialmente significativa a las enfermeras^6^^,^^7^ (el término *enfermera* se utilizará para referirse a profesionales de ambos sexos, siguiendo las recomendaciones del Consejo Internacional de Enfermeras, CIE). Las enfermeras han constituido el grupo más vulnerable, con las tasas más altas de infección por COVID-19, lo que ha provocado un estrés sin precedentes y riesgos para su salud^8^.

En las primeras fases de la pandemia, varios estudios analizaron los efectos emocionales y psicológicos del COVID-19 en personal sanitario^9^^-^^11^. Entre las repercusiones analizadas, los trastornos del sueño, la ansiedad, la depresión y el estrés postraumático fueron las consecuencias negativas más prevalentes experimentadas por las enfermeras durante la pandemia^6^^,^^7^^,^^10^^,^^12^^,^^13^. Uno de esos estudios^10^ analizó la situación de la salud mental de las enfermeras de Navarra, en España, tras la primera ola de la pandemia, concluyendo que el 68% tenía algún grado de depresión, ansiedad, insomnio y estrés postraumático, y un 38% lo tenían en un grado moderado o severo.

Las sucesivas oleadas de la enfermedad, provocadas por diferentes variantes víricas, sometieron a una presión extrema a hospitales y centros de salud, desafiando su capacidad asistencial y poniendo al límite al personal sanitario. La carga de trabajo constante y abrumadora, la escasez de recursos y los síntomas psicológicos derivados de la respuesta a la pandemia, pueden resultar en niveles significativos de *burnout* en los profesionales de la salud que han estado en primera línea frente a la pandemia de COVID-19^14^. Una de las consecuencias de esta situación fue la manifestación de intención de abandono de la profesión por parte de muchas enfermeras. Una revisión sistemática con meta-análisis mostró una tasa de intención de abandono en este colectivo del 31,7% durante la pandemia de COVID-19^13^. Estos resultados, que podrían considerarse previsibles debido a la mayor exposición de las enfermeras a pacientes con COVID-19, así como a la presión constante y a la demanda excesiva durante la pandemia, están respaldados por el hecho de que al menos una de cada cuatro enfermeras experimentaba problemas de salud mental, como ansiedad, estrés, depresión o trastornos del sueño^13^.

El fenómeno de intención de abandono de la profesión enfermera no es nuevo y ya se observaba a nivel global antes de la pandemia^15^^-^^16^. En 2010, Mervi Flinkman y col^15^ realizaron una revisión integradora en la que identificaron que la proporción de enfermeras con intención de abandonar la profesión variaba entre el 4% y el 54%, dependiendo de factores demográficos, laborales y personales. Además, un estudio realizado en China en 2014 reveló que el 5,1% de enfermeras manifestaba la intención de abandonar su trabajo, y que aquellas que trabajaban en entornos laborales favorables eran menos propensas a experimentar altos niveles de agotamiento, insatisfacción laboral e intención de abandono, en comparación con las que se encontraban en entornos laborales deficientes^16^. Por otra parte, el proyecto RN4CAST, publicado en 2013 y en el que participaron 23.159 enfermeras de diez países europeos, analizó los factores relacionados con la intención de abandonar la profesión enfermera en Europa, mostrando que el 9% de las enfermeras tenían la intención de abandonar su profesión, con valores que oscilaban entre el 5% en Países Bajos y España y el 17% en Alemania^17^. El agotamiento o *burnout* mostró una asociación constante con la intención de las enfermeras de abandonar la profesión en los diez países europeos analizados. Aunque los factores del entorno laboral también influyeron en esta decisión, su impacto varió entre países, destacando la importancia de los contextos laborales específicos^17^.

Por lo tanto, el *burnout*^16^^-^^17^, la insatisfacción con el entorno laboral^18^ y la falta de apoyo adecuado, especialmente en las enfermeras recién graduadas^19^, se han identificado como factores que influyen en la intención de abandonar la profesión enfermera. Además, la pandemia de COVID-19 ha podido exacerbar la intención de abandonar la profesión debido al notable agotamiento experimentado por las enfermeras. Así lo ha puesto de manifiesto un estudio realizado en Cataluña, en el que hasta el 53% de las enfermeras encuestadas había reconsiderado en algún momento su situación laboral, y hasta un 69% expresó su intención de abandono^20^.

El abandono de la profesión, motivado por diversos factores como las condiciones laborales precarias, la insatisfacción profesional derivada de una falta de motivación intrínseca^21^, y el impacto de la pandemia de COVID-19^22^, ha agravado el déficit de enfermeras en España. Esta problemática, señalada en el Informe SESPAS publicado en 2024 sobre la escasez de enfermeras en España^23^, también ha sido destacada por la Organización para la Cooperación y el Desarrollo Económico (OCDE), el cual indica que, aunque la pandemia de COVID-19 presentó a las enfermeras como heroínas, también puso de manifiesto las difíciles condiciones laborales y los bajos salarios que enfrentan, lo que ha contribuido a bajos niveles de satisfacción laboral y a un aumento en las intenciones de abandonar la profesión^24^.

Además, un informe político del CIE sobre la escasez global y la retención de enfermeras^25^ destacó que el 90% de los organismos nacionales de enfermería expresaron preocupación por la salud de las enfermeras en el periodo posterior a la pandemia. Sin embargo, son pocos los estudios que han explorado cómo el estado de salud y la carga de trabajo actual de las enfermeras influyen en su compromiso con la profesión enfermera. Por ello, el objetivo de este estudio fue analizar la intención de abandono del puesto de trabajo o la profesión enfermera en Navarra (España) durante la sexta ola de la pandemia por COVID-19 (octubre 2021-marzo 2022)^10^, y la relación existente entre esta intención y el estado de salud mental de las enfermeras y sus características socio-demográficas.

## MATERIAL Y MÉTODOS

*Diseño.* Se realizó un estudio descriptivo y transversal. La población objeto de estudio fueron las enfermeras que desempeñaron su labor en cualquier institución sanitaria de la comunidad foral de Navarra durante la pandemia por COVID-19. Se accedió a 5.700 enfermeras, la población total.

*Muestra.* El tamaño muestral de este estudio se basó en el estudio de Maud M Heinen y col^17^ y se calculó utilizando una prevalencia esperada del 17% para la variable intención de abandono de la profesión, con un nivel de confianza de 0,95 y una precisión del 3% para una población de 5.700 enfermeras. Según estos criterios, se necesitaba una muestra de estudio de 690 enfermeras.

*Muestreo*. Se contactó con el Colegio Oficial de Enfermeras de Navarra con el fin de colaborar y obtener acceso a la totalidad de enfermeras colegiadas. El Colegio envió un correo electrónico a las direcciones de las 5.700 enfermeras colegiadas, invitándoles a participar en el estudio. El correo electrónico incluía una carta redactada por el equipo investigador que proporcionaba detalles sobre el objetivo y las características del estudio, además de presentar los datos de contacto del investigador principal. La primera invitación se envió el 14 de enero de 2022. La respuesta por parte de los participantes fue muy rápida y en cuatro días se alcanzó el tamaño muestral calculado, cerrando así la recogida de datos.

*Recogida de variables*. Se elaboró un cuestionario electrónico para recoger datos sociodemográficos (sexo: hombre/mujer, edad en años, experiencia profesional en años -posteriormente dicotomizada en <10 y en ≥10 años-, lugar de trabajo: hospitalización/atención primaria/centro sociosanitario/gestión) y puesto: enfermera/gestora/otro), y de salud mental (depresión, ansiedad, insomnio y estrés postraumático como resultado de la exposición a un acontecimiento traumático). También se incluyeron dos preguntas relacionadas con la intención de abandonar la profesión enfermera (variable dependiente), basadas en otros estudios^17^^,^^26^^,^^27^. La primera pregunta indagaba si, en caso de ser posible, consideraría dejar su trabajo actual (con opciones de respuesta sí/no), mientras que la segunda pregunta exploraba si contemplaría buscar otro trabajo distinto al de enfermera u otro puesto de enfermera.

Para medir el estado de salud mental de las enfermeras se emplearon cuatro escalas autoadministradas:

- Depresión: Cuestionario de Salud del Paciente (PHQ-9, *Patient Health Questionnaire*)^28^, validado para el contexto español con buenas propiedades psicométricas (resultados equiparables a los de la versión original, demostrando una sensibilidad del 87% y una especificidad del 88%)^29^. Consta de nueve ítems y evalúa la gravedad de la depresión utilizando una escala tipo Likert de 4 niveles (entre 0 y 3 puntos). Las puntuaciones se clasifican en depresión mínima/no depresión (0-4), depresión leve (5-9), depresión moderada (10-14), depresión moderadamente grave (15-19), y depresión grave (20-27).
- Ansiedad: Escala de Trastorno de Ansiedad Generalizada (GAD-7, *Generalized Anxiety Disorder Scale*)^30^, validada para el contexto cultural español y ha mostrado una óptima validez de contenido y una óptima pertinencia en cuanto a la adecuación de los ítems^31^. Está compuesta por siete ítems, con 4 opciones de respuesta puntuables entre 0 y 3, clasifica la ansiedad como mínima/ninguna ansiedad (0-4), ansiedad leve (5-9), ansiedad moderada (10-14) o ansiedad grave (15-21)^30^^-^^31^.
- Insomnio: Índice de Severidad del Insomnio (ISI, Index of Insomnia Severity), instrumento válido y fiable que consta de siete ítems puntuados en una escala de Likert de 0 a 4. La puntuación total se categoriza en insomnio normal/no insomnio (0-7), insomnio subclínico (8-14), insomnio clínico moderado (15-21) e insomnio clínico grave (22-28)^32^.
- Estrés postraumático: Escala de Impacto de Eventos (IES, *Impact of Events Scale*), desarrollada y validada para medir el estrés subjetivo como resultado de la exposición a un evento traumático. El acontecimiento utilizado para este cuestionario fue la aparición del SARS-CoV-2. La escala consta de 22 ítems valorados en una escala de 5 puntos de 0 a 4. Las puntuaciones totales se clasifican como impacto subclínico (0-8), malestar leve (9-25), malestar moderado (26-43) y malestar grave (≥44)^33^.

El cuestionario electrónico se configuró en la plataforma *online* SurveyMonkey y fue evaluado por parte de cuatro integrantes del equipo investigador antes de ser enviado, a fin de garantizar su usabilidad y funcionalidad. Antes de acceder a los ítems, las personas participantes necesitaron leer el consentimiento informado y marcar una casilla que indicaba su participación voluntaria en el estudio. El cuestionario podía ser finalizado y enviado sin la obligación de completar todos los ítems. Sin embargo, únicamente se incluyeron en este estudio los participantes cuyos cuestionarios estaban completamente cumplimentados.

Este estudio se realizó de acuerdo con la Declaración de Helsinki. La investigación fue aprobada por el Comité de Ética, Experimentación Animal y Bioseguridad de la Universidad Pública de Navarra (PI-0020/20). Se solicitó el consentimiento informado de los participantes antes de que cumplimentaran los cuestionarios, y se garantizó el anonimato y la protección de los datos durante el estudio. El estudio siguió las directrices del checklist STROBE para estudios transversales y la *Checklist for Reporting Results of Internet E-Surveys* (CHERRIES).

### Análisis de datos

Se realizó un análisis estadístico descriptivo utilizando frecuencias y porcentajes para las variables cualitativas y medidas de tendencia central (media) y dispersión (desviación estándar, DE) para las variables cuantitativas. Posteriormente, se realizó un análisis inferencial para estimar las principales diferencias en relación con la intención de abandono respecto de las variables sociodemográficas y del estado de salud mental. Para ello, se utilizó la prueba t de Student para variables continuas y la prueba Chi cuadrado (χ^2^) para variables categóricas.

Finalmente, el análisis de regresión logística multivariante permitió conocer la influencia de las distintas variables independientes con la variable dependiente (intención de abandono) cuantificada mediante la *odds ratio* (OR) de cada variable junto con su intervalo de confianza (IC) al 95%. El análisis de los datos se llevó a cabo con el paquete estadístico R, versión 4.2.2 y estableciendo en todos los casos un nivel de significación del 5%.

## RESULTADOS

Se obtuvieron respuestas de 693 participantes, el 92,4% fueron mujeres, con una edad media de 40,5 años (rango: 22-64). La mayoría de enfermeras trabajaban en el ámbito asistencial y en el entorno hospitalario (67,5%), con una media de años trabajados de 17,4 (DE=11,1) (Tabla 1).

**Tabla 1:** Variables sociodemográficas de las participantes en el estudio

Variables	n (%)
*Sociodemográficas*	
Sexo (mujer)	640 (92,4)
Edad (años), *media (DE)*	40,5 (11,3)
*Laborales*	
*Puesto de trabajo*	
Enfermera	651 (93,9)
Gestora	24 (3,5)
Otro	18 (2,6)
*Lugar de trabajo, n (%)*	
Hospitalización	468 (67,5)
Atención Primaria	158 (22,8)
Centro Sociosanitario	48 (6,9)
Gestión	19 (2,7)
Años de experiencia, *media (DE)*	17,4 (11,1)
<10	218 (31,5)
≥10	475 (68,5)

DE: desviación estándar.

Globalmente, entre el 31,2 y el 62,5% de las participantes declaró algún tipo de problema de salud mental clasificado según las puntuaciones medias de los distintos cuestionarios, en el rango de moderado a grave. El 62,5% declaró síntomas de estrés postraumático de moderados a graves. Alrededor de la mitad de las participantes declaró niveles de ansiedad (52%) y de depresión (49%) de moderados a graves. Casi un tercio de las enfermeras participantes sufría de insomnio clínico (31,2%), igualmente calificado como de moderado a grave (Tabla 2).

El 99,7% de participantes respondieron a las preguntas sobre el abandono de la profesión. Un 42,98% respondieron afirmativamente a la pregunta sobre intención de abandono del trabajo actual y, de ellas, el 52% abandonaría la profesión de enfermera, mientras el resto cambiaría de puesto de trabajo (Tabla 3).

**Tabla 2:** Salud mental autodeclarada mediante cuestionarios

Puntuación	Media (DE)
GAD-7 - Ansiedad	10,6 (5,6)
ISI - Insomnio	11 (6,1)
PHQ-9 - Depresión	10,1 (6)
IES - Estrés postraumático	30,4 (16,1)
*Clasificación*	*n (%)*
*Ansiedad*	
Mínima/ninguna	91 (13,1)
Leve	242 (34,9)
Moderada	171 (24,7)
Grave	189 (27,3)
*Insomnio*	
Normal/no insomnio	211 (30,4)
Subclínico	266 (38,4)
Clínico moderado	189 (27,3)
Clínico grave	27 (3,9)
*Depresión*	
Mínima/no depresión	140 (20,2)
Leve	213 (30,7)
Moderada	177 (25,5)
Moderadamente grave	111 (16,0)
Grave	52 (7,5)
*Estrés postraumático*	
Subclínico	92 (13,3)
Leve	168 (24,2)
Moderado	263 (38,0)
Grave	170 (24,5)

DE: desviación estándar

**Tabla 3:** Intención de abandono de la profesión

*Intención de dejar el trabajo actual*	*n (%)*
Sí	297 (42,98)
No	394 (57,0)
*Intención de buscar trabajo nuevo*	*n (%)*
De enfermera en otro sitio	143 (48,0)
No como enfermera	155 (52,0)

**Figura 1:**
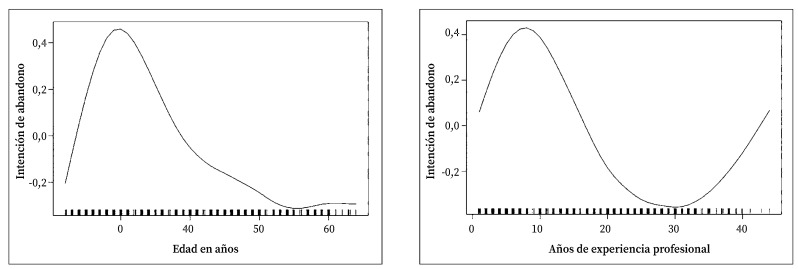
Intención de abandono de la profesión según edad y años de experiencia.

Quienes declararon intención de abandonar el puesto de trabajo tenían una edad media significativamente inferior (39,11 años frente a 41,57; p=0,005). Coincidiendo con este resultado, las enfermeras con menor número de años trabajados declararon más frecuentemente la intención de abandonar la profesión (p=0,002) (Fig. 1).

También se encontraron diferencias significativas en la intención de abandonar según el puesto y el lugar de trabajo. Un mayor porcentaje de enfermeras asistenciales mostraron intención de abandonar en comparación con gestoras u otros puestos. Las enfermeras que trabajaban en hospitalización también mostraron una mayor intención de abandono de la profesión (Tabla 4).

Respecto al estado de salud mental, las enfermeras con intención de abandonar su profesión presentaron puntuaciones medias significativamente mayores en todas las variables estudiadas: ansiedad, insomnio, sueño y estrés postraumático (Tabla 4).

**Tabla 4:** Intención de abandono según variables demográficas, sociolaborales y de salud mental

Variables	Intención de abandono		p
	No (n=394)	Sí (n=297	
Sexo (masculino), n (%)	34 (8,6)	18 (6,1)	0,262
Edad (años), media (DE)	41,57 (11,43)	39,11 (11,10)	0,005
*Puesto de trabajo, n (%)*			*0,002*
Enfermera asistencial	360 (91,4)	289 (97,3)	
Gestora	17 (4,3)	7 (2,4)	
Otro	17 (4,3)	1 (0,3)	
*Lugar de trabajo, n (%)*			*0,009*
Atención Primaria	72 (18,3)	85 (28,6)	
Centro socio sanitario	26 (6,6)	22 (7,4)	
Gestión	13 (3,3)	6 (2,0)	
Hospitalización	283 (71,8)	184 (62,0)	
Años trabajados, media (DE)	18,50 (11,11)	15,83 (10,96)	0,002
*Salud mental, media (DE)*			
Total ansiedad	8,65 (5,10)	13,22 (5,13)	<0,001
Total insomnio	9,34 (5,72)	13,19 (6,00)	<0,001
Total depresión	8,06 (5,44)	12,73 (5,72)	<0,001
Total estrés postraumático	25,07 (15,36)	37,57 (14,02)	<0,001

DE: desviación estándar.

Por último, se realizó un análisis de regresión logística múltiple incorporando las variables sociodemográficas (sexo, edad, lugar de trabajo, y años trabajados) y el estado de salud mental mediante las puntuaciones de los instrumentos de medida de ansiedad, depresión, insomnio y estrés postraumático. Los resultados revelaron que la ansiedad y el estrés postraumático eran predictores significativos de la intención de abandonar la profesión, indicando que, por cada punto adicional en la puntuación total GAD-7 (OR=1,09; IC 95%: 1,03-1,15; p=0,001) e IES (OR=1,03; IC 95%: 1,01-1,05; p=0,004), la probabilidad de abandono aumentaba en un 9% y un 3%, respectivamente. Tener menos de 10 años trabajados incrementó un 77% la probabilidad de que la enfermera deseara abandonar la profesión (Tabla 5).

**Tabla 5:** Efecto de las variables sociodemográficas y de salud mental sobre la intención de abandono de la profesión (regresión logística múltiple)

Variable	OR	IC95%	p
*(Intercept)*	*0,06*	*0,01 - 0,22*	*<0,001*
Puntuación total GAD-7	1,09	1,03 - 1,15	0,001
Puntuación total ISI	1	0,96 - 1,04	0,937
Puntuación total PHQ-9	1,03	0,98 - 1,09	0,209
Puntuación total IES	1,03	1,01 - ,05	0,004
Sexo (hombre/mujer)	0,94	0,48 - 1,80	0,849
Edad	1,01	0,99 - 1,04	0,421
Lugar de trabajo Centro socio sanitario	1,09	0,53 - 2,26	0,811
Lugar de trabajo Gestión	0,71	0,22 - 2,08	0,543
Lugar de trabajo Hospitalización	0,85	0,56 - 1,30	0,452
Años trabajados (<10/≥10)	1,77	1,00 - 3,13	0,049

OR: *odds ratio*; IC95%: intervalo de confianza al 95%.

## DISCUSIÓN

Este estudio determinó que en Navarra (España) y tras la sexta ola de la pandemia por COVID-19 (octubre 2021-marzo 2022), un 42,98% de las enfermeras dejarían su trabajo si pudieran. Además, más de la mitad de estas enfermeras habían considerado en algún momento dejar la profesión y buscar trabajo en otro campo no relacionado con la sanidad o su labor actual, aspecto también destacado en otros contextos^25^.

La creciente carga laboral ha tenido un impacto significativo tanto en el bienestar físico como emocional de las enfermeras. Además, este porcentaje de intención de abandono es considerablemente más alto en comparación con los hallazgos de estudios previos realizados durante las etapas iniciales de la pandemia. Por ejemplo, en el estudio de Rosanne Raso y col^26^, solamente un 11% de las enfermeras encuestadas manifestaron su intención de abandonar su puesto de trabajo. Además, el metaanálisis llevado a cabo por Fadime Ulupınar y Yasemin Erden^13^ arrojó una estimación global del 31,7% para la intención de dejar la profesión entre las enfermeras durante la pandemia de COVID-19.

El presente estudio destaca que la ansiedad y el estrés postraumático desempeñaron un papel clave como factores determinantes para la intención de abandonar la profesión. También moderados niveles de ansiedad y de estrés contribuyeron de manera significativa a la intención de abandono. Hay abundante evidencia del gran impacto negativo en la salud mental de las enfermeras que han trabajado durante la pandemia^6^^,^^7^^,^^26^^,^^27^^,^^34^^-^^38^. En otro estudio realizado con enfermeras en Navarra, llevado a cabo entre abril y mayo de 2021, se identificó un 38% de alteraciones moderadas y severas en la salud mental de las enfermeras^10^.

La asociación entre el estrés postraumático y la intención de abandono de la profesión enfermera también se ha identificado en el contexto de otros brotes de virus respiratorios graves, tales como el Síndrome Respiratorio de Oriente Medio (MERS)^39^. Por tanto, este patrón resalta la importancia de que las intervenciones destinadas a mejorar el bienestar psicológico en el lugar de trabajo se centren particularmente en reducir la ansiedad y el estrés postraumático de las enfermeras, con el fin de mejorar su retención y reducir su deseo de abandono. Heeja Jung y col^39^ describieron cómo el apoyo social en el lugar de trabajo, específicamente el apoyo por parte de supervisores, tiene un efecto amortiguador del impacto del estrés postraumático en las intenciones de abandono de las enfermeras.

Otros estados de salud mental como el insomnio y la depresión no mostraron un efecto significativo en la intención de abandono cuando se ajustaba por otras variables, aunque pueden ser relevantes en otros contextos.

La experiencia profesional (y especialmente menos de 10 años trabajados) también resultó otro factor relevante asociado con la intención de abandonar la profesión. Esto sugiere que las enfermeras con menos experiencia pueden estar menos capacitadas o tener menos herramientas para manejar los factores estresantes y las demandas del trabajo, especialmente los primeros años de actividad profesional. Esto podría explicarse por el hecho de que las enfermeras de mayor edad pueden enfrentar inquietudes adicionales y diferentes a las de sus colegas más jóvenes, como las responsabilidades familiares y sociales que se suman a su labor profesional^40^. Además, ser mujer se asociaba con un peor estado de salud mental, un aspecto que también ha sido observado en otros estudios^37^^,^^41^^,^^42^. En este estudio, el 92,4% de la muestra son mujeres, un resultado consistente con la composición predominante de la profesión enfermera, que es mayoritariamente femenina. Factores como el sexo, la edad y el lugar de trabajo, aunque mostraron algunas diferencias en los análisis descriptivos, no se mantuvieron como predictores significativos en el análisis multivariado. Esto podría implicar que, al considerar múltiples factores simultáneamente, las diferencias observadas inicialmente son explicadas mejor por otros factores más directamente relacionados, como los problemas de salud mental y los años trabajados.

Considerando los hallazgos de este estudio, las políticas y programas de apoyo deben centrarse en proporcionar recursos adecuados para manejar estos factores psicológicos, así como en ofrecer un entorno laboral que apoye a las enfermeras menos experimentadas. La implementación de programas de mentoría, capacitación en manejo del estrés y apoyo psicológico puede ser crucial para mejorar la estabilidad en la profesión y reducir la intención de abandono. Resulta imperativo implementar estrategias que aborden la alarmante escasez de enfermeras en todo el mundo. En primer lugar, es fundamental abordar las necesidades de salud mental de las enfermeras cuyo bienestar emocional está siendo afectado, trabajando con recursos que permitan mediar sobre la vivencia y causas que generan ansiedad y estrés postraumático. Además, es esencial crear entornos laborales saludables que eviten la sobrecarga de trabajo y el *burnout*, promoviendo una ratio de enfermeras adecuada para garantizar la seguridad y la eficacia de los cuidados. Para lograr esto, el sistema de contratación debe evitar los contratos precarios, reducir las rotaciones frecuentes entre diferentes servicios para una misma enfermera y favorecer contratos de larga duración^43^. También los entornos de atención sanitaria deben incentivar la sensación de pertenencia del personal al equipo, fomentar su crecimiento profesional y personal, y reconocer su labor. Como se indica en el trabajo de Constanze Leineweber y col^44^, mejorar el entorno laboral y ofrecer mayor flexibilidad horaria podrían ser estrategias prometedoras para aumentar la retención de enfermeras en sus lugares de trabajo.

Por último, se debe considerar que este estudio se centra en enfermeras que trabajan en Navarra, por lo que sus resultados podrían no ser generalizables a otras regiones o contextos. Sin embargo, un estudio cualitativo realizado con una muestra de catorce enfermeras en otra comunidad autónoma española, Cataluña, ha investigado los factores que influyen en la intención de abandonar la profesión enfermera tras la pandemia, concluyendo que, aunque las enfermeras experimentaron impactos emocionales significativos, mostraron dedicación y resiliencia. Su decisión de continuar en la profesión estuvo influida por factores como el sentido de responsabilidad, el sentimiento de culpa y la estabilidad económica^45^. Todo esto pone de manifiesto la necesidad de continuar realizando estudios adicionales para evaluar las estrategias de gestión adecuadas para tratar y prevenir los trastornos de salud mental entre los trabajadores sanitarios, así como estudios que abarquen intervenciones de gestión que permitan mejorar el clima laboral y analizar la filiación de los trabajadores tras ello, recogiendo datos en diferentes contextos y en la actual etapa de postpandemia.

Los hallazgos de este estudio revelan que las enfermeras han presentado niveles altos de intencionalidad de abandono de la profesión, siendo el estado emocional (nivel moderado de ansiedad y estrés postraumático) un factor determinante de riesgo de abandono de la profesión. Además, las enfermeras con menos experiencia profesional muestran una mayor intención de abandonar la profesión. Estos hallazgos destacan la necesidad de implementar estrategias efectivas para mejorar las condiciones laborales, promover el bienestar mental de las enfermeras y reducir el riesgo de abandono profesional, especialmente en contextos de crisis sanitaria.

## Data Availability

Se encuentran disponibles bajo petición al autor de correspondencia.
